# SERPINE1 maintained expression by NR4A1 promotes invasion and migration of glioblastoma in hypoxic microenvironment

**DOI:** 10.3389/fonc.2025.1750546

**Published:** 2026-01-09

**Authors:** Zhennan Tao, Yi Sun, Yimuran Yilamu, Bo Xu, Chen Yu, Hao Zhang, Lingyun Wu, Wanli Yu, Yuxiang Dai

**Affiliations:** 1 Department of Neurosurgery, Nanjing Drum Tower Hospital, Affiliated Hospital of Medical School, Nanjing University, Nanjing, Jiangsu, China; 2 Neurosurgical Institute, Nanjing University, Nanjing, Jiangsu, China; 3 Department of Neurosurgery, The Second Affiliated Hospital, Jiangxi Medical College, Nanchang University, Nanchang, Jiangxi, China; 4 Jiangxi Province Key Laboratory of Neurological Diseases, Nanchang University, Nanchang, Jiangxi, China; 5 JXHC Key Laboratory of Neurological Medicine, Nanchang University, Nanchang, Jiangxi, China; 6 Institute of Neuroscience, Nanchang University, Nanchang, Jiangxi, China

**Keywords:** glioblastoma, hypoxic microenvironment, invasion, NR4A1, SERPINE1

## Abstract

**Introduction:**

The activation of epithelial mesenchymal transition (EMT) characteristics in GBM cells is the main factor leading to this invasion and migration. Serpin family E member 1 (SERPINE1) encodes plasminogen activator inhibitor-1 (PAI-1), which plays a key role in regulating the extracellular matrix and is closely related to tumor progression and metastasis, especially in gliomas. However, the exact molecular mechanism of its role in GBM is still unclear.

**Methods:**

In this study, we evaluated the targeted therapeutic value of SERPINE1 through bioinformatics analysis. Study the effect of SERPINE1 inhibition on GBM cell proliferation and invasion using *in vitro* and *in vivo* models. Observe the EMT characteristics of hypoxia induced GBM cells and analyze the interaction between NR4A1 and SERPINE1 through molecular biology methods.

**Results:**

Our research results indicate that GBM cells cultured in a low oxygen microenvironment have higher invasiveness, characterized by the activation of EMT markers. Inhibition of SERPINE1 *in vitro* can significantly reduce the proliferation and invasion ability of GBM cells. Further *in vivo* experiments have confirmed that targeting SERPINE1 can effectively inhibit the growth of GBM, reduce tumor size and proliferation in mouse models. In addition, we found that SERPINE1 can bind to NR4A1 and may have an interaction.

**Conclusion:**

This study provides new insights into the molecular mechanisms underlying the progression of GBM, emphasizing the role of SERPINE1 and its interaction with NR4A1 in promoting EMT and tumor invasion. Inhibiting the expression of SERPINE1 in GBM cells can prevent cell invasion, providing a potential strategy for the treatment of GBM.

## 1 Introduction

Glioma is the most common primary intracranial tumor. According to the latest statistics from the American Brain Tumor Registry, glioblastoma (GBM, WHO grade IV) has the highest incidence among primary malignant central nervous system tumors, accounting for 47.7% (1, 2). Currently, adjuvant therapies following glioma surgery are diverse, but their overall effectiveness is limited, and the incidence is on the rise. The 1-year and 5-year survival rates for adult high-grade gliomas are approximately 30% and 13%, respectively, while the median survival time for anaplastic gliomas and glioblastomas is about 2–3 years and 1 year, respectively (3). The invasive growth of gliomas is its most prominent biological feature, leading to tumor migration and recurrence, and is a major cause of death in glioma patients (4). Therefore, regulating the invasion of glioma cells has been a key focus of glioma research, and identifying new targets to effectively control their invasive growth is crucial for slowing the progression or recurrence of gliomas.

The invasion of GBM is the result of continuous interaction between tumor cells and their microenvironment (5, 6). The tumor microenvironment consists of various extracellular matrix (ECM) components, including soluble factors such as fluids, chemokines, and cytokines, as well as cells like neurons, astrocytes, oligodendrocytes, endothelial cells, and immune cells. The ECM, due to its varying components, creates different cellular niches and promotes migration and invasion in distinct ways. One of the most prominent examples of a pathological niche in the brain is the hypoxic microenvironment (7). Hypoxia stimulates the expression of hypoxia-inducible factors HIF1α and HIF2α, which facilitate the acquisition of an invasive phenotype by tumor cells (8). An acidic environment also stimulates HIF function and activates matrix metalloproteinases, which, in turn, induces similar mechanisms by degrading the extracellular matrix. Tumor cells in different microenvironments exhibit distinct invasion and migration characteristics, with GBM cells in the hypoxic microenvironment being more prone to acquiring invasive traits (9).

Hypoxia can induce cellular phenotypic changes and, in collaboration with other pathways, promote EMT, thereby enhancing the invasive characteristics of tumors. Current studies have found that several transcription factors can induce EMT. These transcription factors include zinc finger-binding transcription factors Snail1 and Snail2 (also known as Slug), as well as other basic helix-loop-helix (bHLH) factors such as E-box binding zinc finger proteins 1 (ZEB1), ZEB2, and Twist (10). These proteins bind to the promoter regions of cell adhesion-related genes and inhibit their transcription, which is a key initiating step of EMT (11). Hypoxia suppresses the activity of prolyl hydroxylases such as PHD2 and PHD3, which, under normoxic conditions, catalyze and degrade HIF-1α. HIF-1α is also a transcription factor that induces the expression of several EMT-related genes, including TGF-β, Snail1, TWIST, and LOX (12, 13). HIF-1α can directly bind to the hypoxia response element in the TWIST1 promoter (14). In hypoxia-induced EMT, HIF-1α increases the abundance of HDAC3. HDAC3 binds to the promoter of CDH1 and cooperates with Snail1 to suppress their transcription. Additionally, HDAC3 mediates the formation of histone methyltransferase complexes, which are necessary for the expression of mesenchymal cell markers such as vimentin and N-cadherin (15). Although much research has been conducted on EMT, breakthroughs in targeted therapies directly regulating EMT have not yet been achieved.

With the development of high-throughput sequencing technologies in recent years, abnormal expression of SERPINE1 has been detected in various cancers, and its role in tumors has garnered significant attention (16). It has been reported that SERPINE1 can induce tumor migration, invasion, and angiogenesis, thereby promoting tumor progression and metastasis (17). For example, it has been reported that SERPINE1 is elevated in gastric adenocarcinoma tissues, and its upregulation can enhance tumor cell invasion and proliferation by regulating epithelial-mesenchymal transition (EMT) (18, 19). Additionally, SERPINE1 has been identified as a regulator of glioblastoma cell dissemination, and downregulating SERPINE1 can limit glioma cell proliferation and invasion (20). Furthermore, it has been reported that ACT001, a direct inhibitor of SERPINE1, suppresses glioma cell proliferation, migration, and invasion by inhibiting the PI3K/AKT pathway (21). Studies have explored its expression characteristics and biological functions in low-grade glioma cohorts, finding that SERPINE1 can not only serve as a prognostic biomarker but also as a potential therapeutic target for glioma (22). However, the specific molecular mechanisms by which SERPINE1 induces these phenotypic changes in glioma remain unclear.

In this study, we evaluated the association between SERPINE1 and NR4A1 in the context of GBM invasion under a hypoxic microenvironment. Our results show that inhibiting SERPINE1 reduced EMT transition and in GBM and demonstrated tumor growth inhibition in an orthotopic implantation tumor model. Further investigation revealed a binding relationship between NR4A1 and SERPINE1, with their expression levels being positively correlated. Therefore, targeting SERPINE1 could be a promising therapeutic strategy for preventing GBM progression.

## 2 Materials and methods

### 2.1 Cell culture

The GBM cell line U251 were obtained from the Cell Bank of the Chinese Academy of Sciences. We confirm the recent STR analysis and mycoplasma detection of U251 cell. The cells were cultured in DMEM medium supplemented with 10% fetal bovine serum (FBS, Gibco, US).

### 2.2 Data collection and analysis

RNA sequencing (RNA-seq) transcriptional data and clinical information of glioma were downloaded from The Cancer Genome Atlas (TCGA) database (https://cancergenome.nih.gov/) and the Chinese Glioma Genome Atlas (CGGA) database (https://www.cgga.org.cn/). The bar plot of differential gene expression, clinical prognosis line plot, heatmap, and correlation analysis plot were generated using R programming (version 4.4.2). The PPI protein network analysis was performed by incorporating these mRNAs into the PPI network using the STRING database (https://string-db.org/) with a confidence score >0.8. The PPI network was visualized using Cytoscape (version 3.8.1).

### 2.3 Lentiviral shRNA transfection

Lentiviral shRNAs targeting the SERPINE1 gene were generated using the GV112 vector (hU6-MCS-CMV-Puromycin; GeneChem, China). The SERPINE1 overexpression lentivirus was constructed using the GV492 vector (Ubc-MCS-3FLAG-CBh-gcGFP-IRES-puromycin; GeneChem, China). Cells were transfected with either scrambled control or shRNA-expressing lentiviral vectors following the manufacturer’s recommendations. After infection, stable cell clones transfected with shRNA-expressing constructs were selected using puromycin solution. Cells were harvested 48 hours post-transfection for subsequent experiments.

### 2.4 Wound healing assessment and trans-well assay

For wound healing assessment, cells from different treatment groups were seeded into 6-well plates and incubated overnight, with one group cultured under hypoxic conditions (5% O2). The following day, after cells adhered to the plate, a scratch assay was performed. Migration distance was calculated at 24 and 48 hours using GraphPad. For the Transwell invasion assay, to evaluate cell invasion ability, 5 × 10^4^ tumor cells were seeded into the upper chamber of a Transwell insert coated with matrigel and cultured in serum-free medium. The same incubation conditions were used as described above. The lower chamber contained 500 µL complete medium with 10% FBS to induce cell invasion. After 24 hours of incubation, cells that migrated through the membrane were stained with 0.2% crystal violet and photographed under a microscope.

### 2.5 Co immunoprecipitation

Total cell lysates were extracted on ice using RIPA lysis buffer supplemented with protease inhibitors. A small portion of the lysate was retained as input and centrifuged at 12,000 × g for 10 minutes. The remaining cell extract was incubated overnight at 4°C with NR4A1 primary antibody (Abcam, ab283264), and mouse IgG or rabbit IgG antibodies were used as negative controls. The following day, magnetic beads were added to the protein lysate and incubated for 2 hours at 4°C. Afterward, the peak-enriched magnetic beads were washed with PBS, and the bound SERPINE1 (CST #49536) expression was analyzed by western blotting.

### 2.6 Western blot and immunofluorescence

The total protein was extracted from cells using pre-chilled RIPA buffer containing 1% protease and phosphatase inhibitor mixture. Protein concentration was measured using the BCA assay (Sigma, Catalog No. QPBCA). The samples were subjected to SDS-PAGE and transferred onto PVDF membranes. The membranes were blocked with 5% milk in TBST solution and incubated overnight with the primary antibody at 4°C. Subsequently, all membranes were incubated with mouse/rabbit IgG secondary antibody at room temperature for 1 hour. Imaging was performed on a FluorChem E system (Cell Biosciences). For immunofluorescence, after cell adhesion, the cells were fixed with 4% paraformaldehyde, blocked with 5% BSA, the primary antibody was added, and the samples were placed in a wet box at 4°C overnight. The next day, the samples were incubated with fluorescent secondary antibodies and sealed with DAPI mounting medium. The coverslips with attached cells were placed on adhesive slides. Finally, images were captured using a confocal microscope. The primary antibodies used included E-cadherin (CST #3195), N-cadherin (CST #14215), and β-Tubulin (CST #2146).

### 2.7 H&E staining

The glass slide is deparaffinized and rehydrated. Next, the slide is subjected to nuclear staining, followed by H&E staining using the H&E staining kit (Solarbio, China). Images are captured using a VANOX microscope (Olympus, Japan).

### 2.8 Intracranial xenograft model in nude mouse

Female BALB/c nude mice (4–5 weeks old, weighing 15–17 grams) were obtained from the SPF (Beijing) Biotechnology Co., Ltd. animal center and bred under specific pathogen-free conditions. U251 and U251 (shSERPINE1) cells, induced with luciferase, were stereotactically injected into the brains of the nude mice, with coordinates 1 mm anterior to the bregma on the right hemisphere, 2 mm lateral, and a depth of 5 mm. After a 12-hour light/dark cycle, all mice were kept under appropriate temperature and humidity conditions. They were fed a standard diet and had free access to water. Bioluminescence imaging (BLI) was used to monitor intracranial tumors at specified time points. Tumor growth was observed using BLI at days 7, 14, 21, and 28. BLI measurements of intracranial tumors were taken *in vivo* (n=3). Mice were euthanized at designated time points following tumor implantation or onset of neurological symptoms. Upon the death of all mice, survival times were recorded, and tissues were carefully extracted and fixed in 10% formalin for H&E staining. All experimental mice were euthanized via cervical dislocation following deep anesthesia induced by isoflurane inhalation. Anesthesia was administered using 5% isoflurane in 100% oxygen at 1 L/min flow rate for induction (3–5 minutes), maintained at 2-3% isoflurane until loss of consciousness. Death was confirmed by absence of corneal reflex and respiratory arrest for >60 seconds. All animal procedures were approved by the Animal Care and Use Committee of the Affiliated Drum Tower Hospital, Nanjing University Medical School.

### 2.9 Statistical analysis

The bar chart represents the mean standard deviation from at least three experimental replicates. The number of repeated experiments involved is n=3. Most of the experiments were statistically analyzed using Student’s t test. The data were analyzed by graphpad prism 6. Significance of p values were set at ^NS^P > 0.05, *P < 0.05, **P < 0.01, ***P< 0.001.

## 3 Results

### 3.1 SERPINE1 is a key factor in regulating the invasion of GBM cells

**Figure 1:**
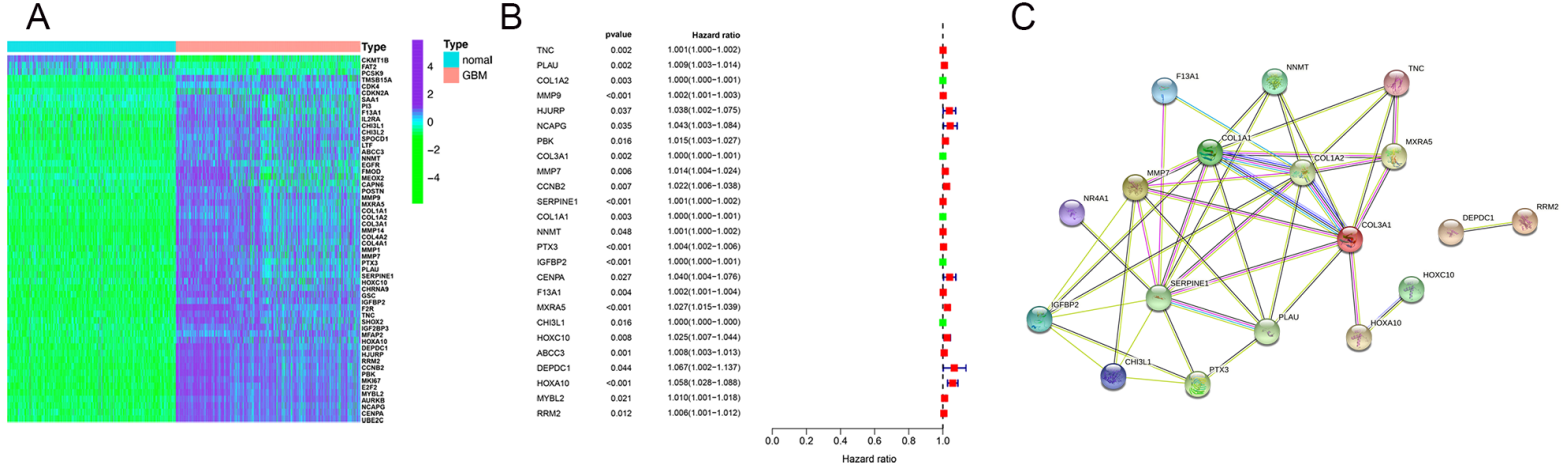
SERPINE1 is a key regulator of GBM cell invasion. EMT gene sets were obtained from the EMTome database. After fitting the gene expression data and patient clinical prognosis data from the TCGA and CGGA databases, **(A)** the expression levels of EMT gene sets in normal tissue samples and GBM patient samples were compared, and a heatmap was plotted; **(B)** genes with significant expression differences were subjected to ROS prognosis risk scoring, further screening high-risk genes; **(C)** the final selected gene set was analyzed using PPI protein network analysis.

To identify precise regulatory sites of EMT in GBM, we analyzed EMT-related genes using the EMTome database (http://www.emtome.org/), which provided a list of 3,606 EMT-associated genes. By combining expression profiles and survival data from GTEx, TCGA, and CGGA, we compared the expression differences between normal tissues and GBM tissues. ROS risk prediction and clinical prognosis analysis were conducted, leading to the identification of approximately 25 genes potentially associated with EMT in GBM (**Figures 1A, B**). Furthermore, to determine which of these genes play a central regulatory role in EMT, we performed PPI (protein-protein interaction) network analysis on the proteins encoded by these genes. The proteins at the regulatory core were found to be COL1A1, COL1A2, COL3A1, and SERPINE1 (**Figure 1C**). Since brain parenchyma lacks components such as collagen, COL1A1, COL1A2, and COL3A1 are not suitable for studying GBM. Therefore, we first identified SERPINE1 as a key regulatory site of EMT, suggesting that SERPINE1 may participate in GBM invasion through regulating EMT.

### 3.2 Clinical prognosis analysis and biological characteristics of SERPINE1

**Figure 2:**
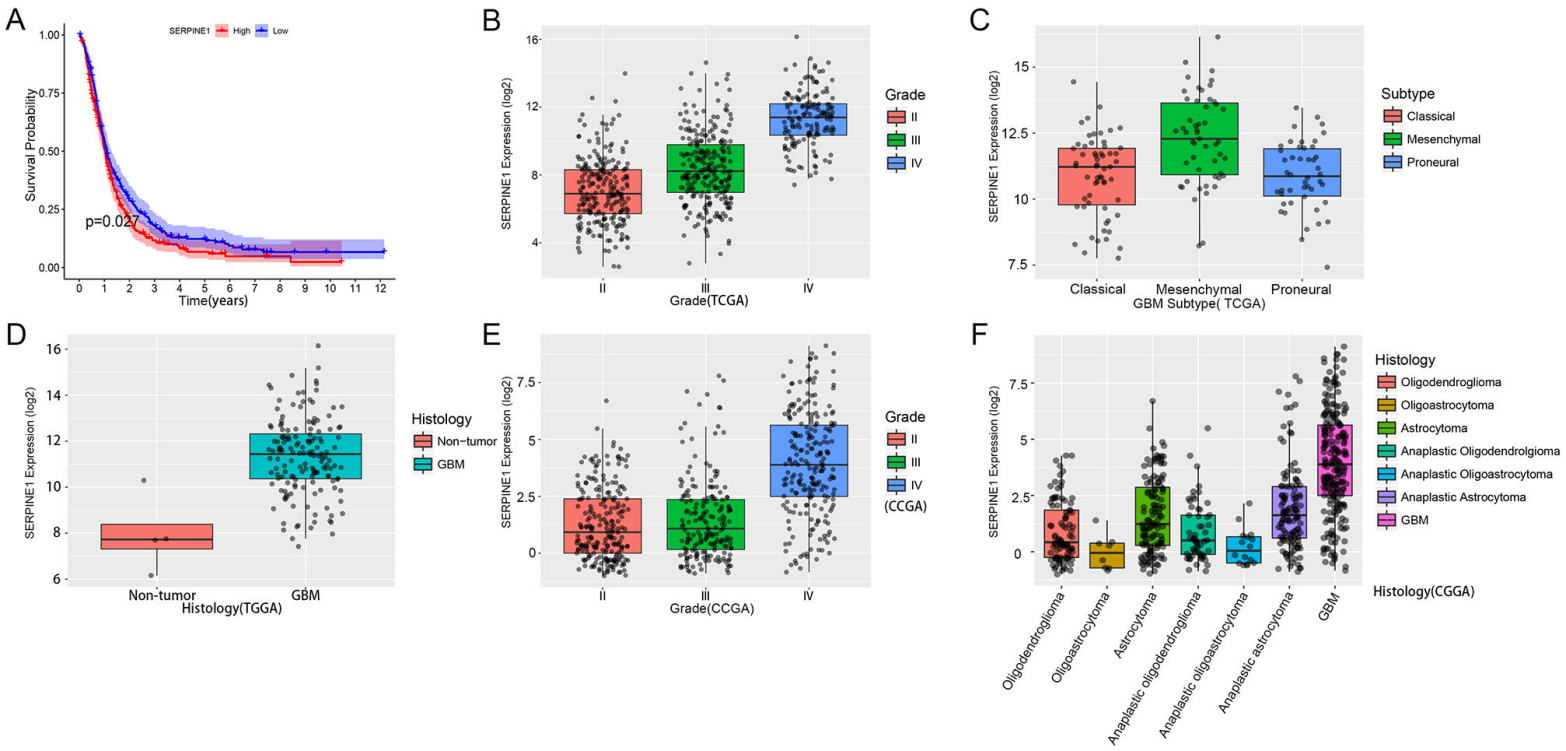
Expression characteristics and prognostic analysis of SERPINE1 in glioma. **(A)** Combined data from TCGA and CGGA were used for survival analysis of glioma patients to assess the correlation between SERPINE1 expression and overall survival (OS) in glioma patients. The median of the data was used to differentiate between low and high expression of SERPINE1 in patient samples; **(B, E)** TCGA and CGGA datasets were used to evaluate the expression of SERPINE1 across different WHO grades; **(C)** Expression of SERPINE1 in various molecular subtypes of GBM in the TCGA database; **(D)** Expression of SERPINE1 in normal tissue versus GBM tissue in the TCGA database; **(F)** Expression of SERPINE1 in different pathological classifications in the CGGA database.

Through analysis of data from the TCGA and CGGA databases, we found that the expression of SERPINE1 is higher in GBM compared to normal tissue and is associated with glioma grading. SERPINE1 expression is highest in GBM and is present in the unique proteomic features of the GBM mesenchymal subtype, which corresponds to the low survival rate, invasiveness, and drug-resistant phenotype in GBM (**Figures 2B–F**). In the prognosis analysis, high levels of SERPINE1 expression were closely linked to poor survival rates in glioma patients (**Figure 2A**). Therefore, SERPINE1 serves as a powerful prognostic marker for GBM and may play a key role in GBM invasion through mechanisms that are yet to be discovered.

**Figure 3:**
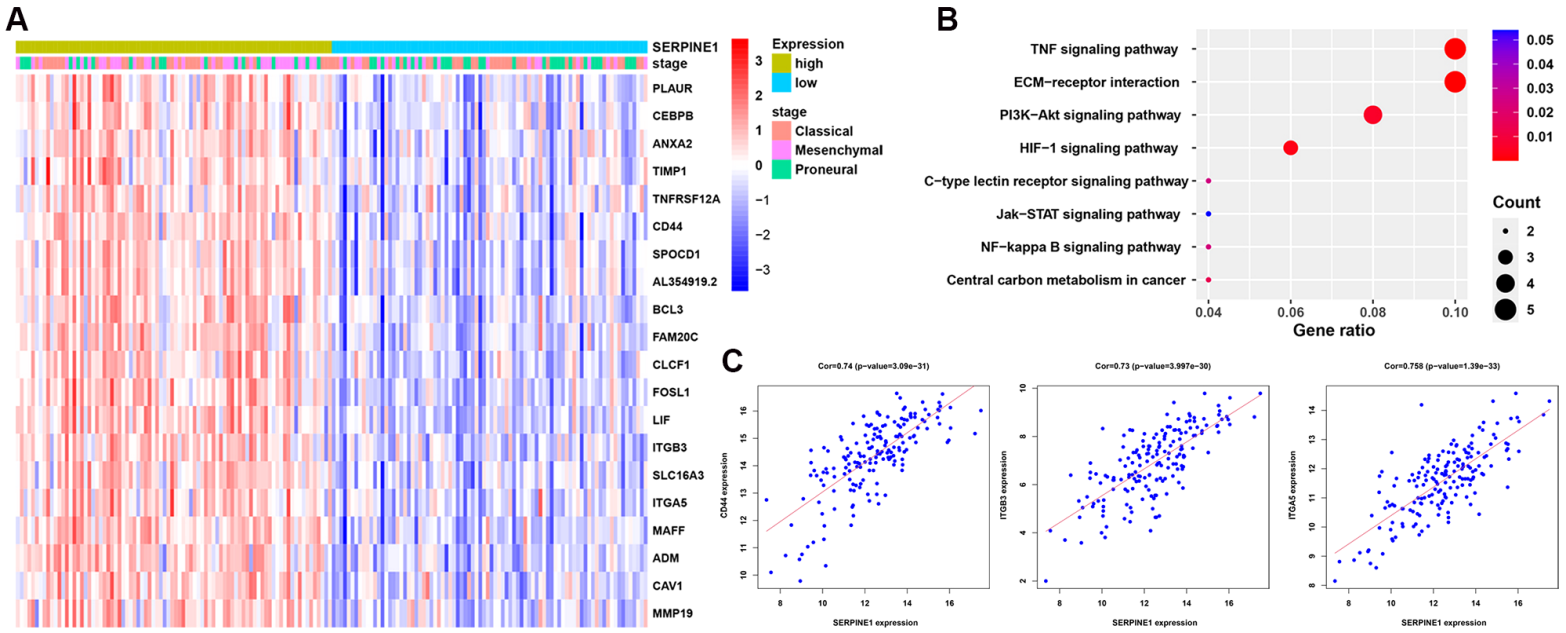
Heatmap of SERPINE1-related genes and gene correlations. **(A)** Heatmap visualizing genes highly correlated with PFKFB4 based on PFKFB4 expression levels in GBM cell subtypes, including classical, proneural, and mesenchymal types. **(B)** KEGG analysis of the top 50 genes with high correlation. **(C)** Gene expression correlation with SERPINE1 that may indicate potential interactions.

To further explore the molecular mechanisms potentially involved in SERPINE1, we performed expression analysis on the top 20 genes most closely related to SERPINE1 expression. We constructed a heatmap based on SERPINE1 expression levels and GBM subtypes and conducted KEGG analysis to identify potential regulatory pathways associated with SERPINE1. We found that genes related to cell invasion and migration, such as CD44, ITGB3, and ITGA5, showed high correlation with SERPINE1 (**Figures 3A–C**).

### 3.3 SERPINE1 regulates EMT in GBM cells

**Figure 4:**
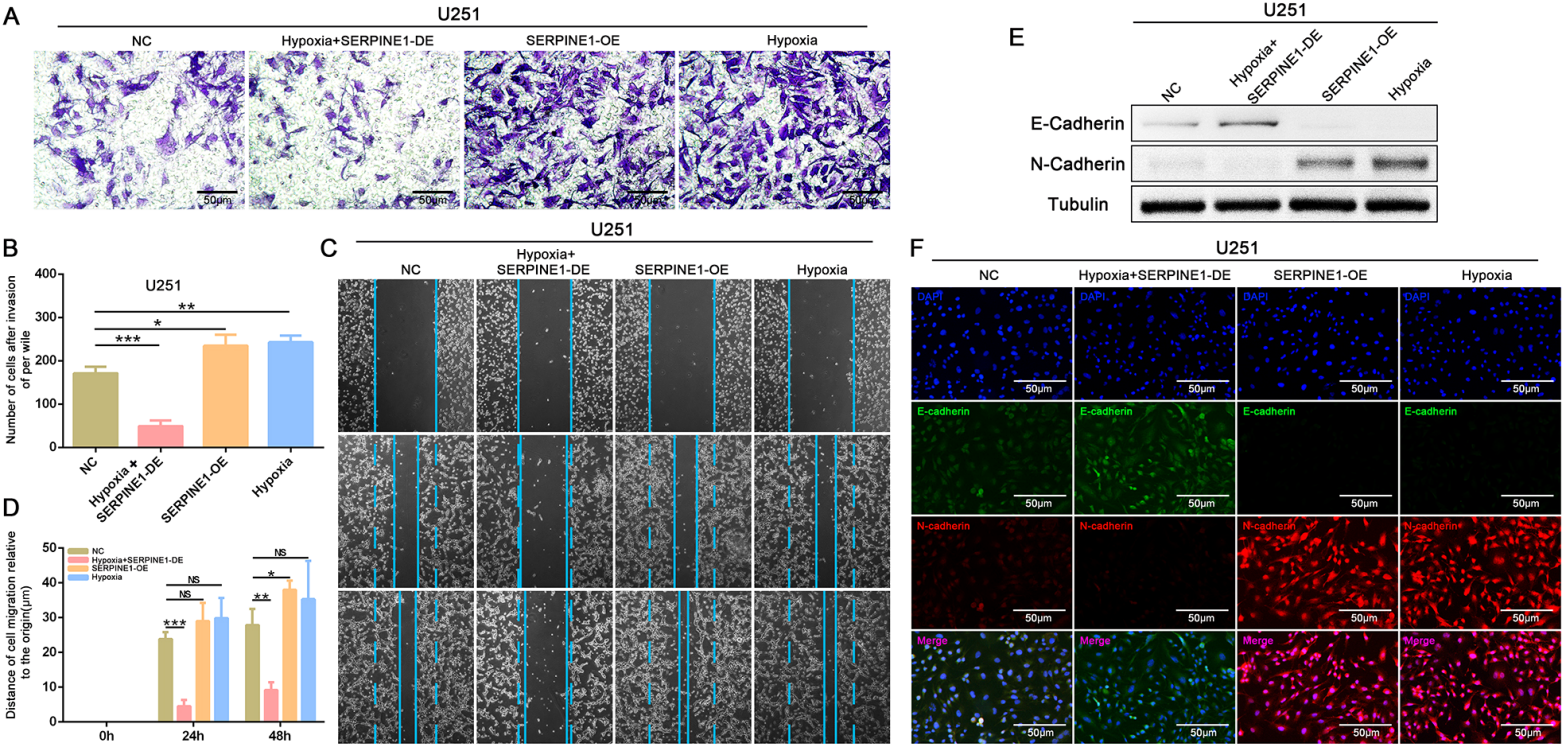
SERPINE1 promotes EMT characteristics in GBM cells. The experimental groups were as follows: NC group, SERPINE1 knockdown group, SERPINE1 overexpression group, and hypoxia treatment group. **(A)** Transwell assay was used to detect the invasion efficiency of GBM cells; **(B)** The number of cells that invaded through the Matrigel in each experimental group was counted; **(C)** Scratch assay was performed to assess the migration ability of GBM cells in each group; **(D)** The relative distance from the initial position of GBM cell migration in each group was measured; **(E)** Western blot analysis was used to detect changes in the expression of E-cadherin and N-cadherin; **(F)** Immunofluorescence was used to detect the expression of E-cadherin and N-cadherin, scale bar = 50 μm. Data are shown as the mean ± S.D. n = 3, *P < 0.05,**P < 0.01, ***P < 0.001, Student’s t-test.

We evaluated the effect of SERPINE1 on the invasion and migration abilities of GBM cells. The results showed that under conditions of SERPINE1 overexpression and hypoxia, the invasion and migration abilities of GBM cells were enhanced, while knockdown of SERPINE1 significantly reduced these abilities (**Figures 4A–D**). Protein level analysis revealed that inhibition of SERPINE1 decreased the EMT characteristics of GBM cells, as evidenced by an increase in E-cadherin expression and a decrease in N-cadherin expression. Under hypoxic conditions and SERPINE1 overexpression, EMT characteristics were enhanced (**Figures 4E, F**). Therefore, we concluded that SERPINE1 promotes the EMT characteristics of GBM cells.

### 3.4 Knock-down of SERPINE1 inhibited the infiltration of GBM cells in *in vivo* models

**Figure 5:**
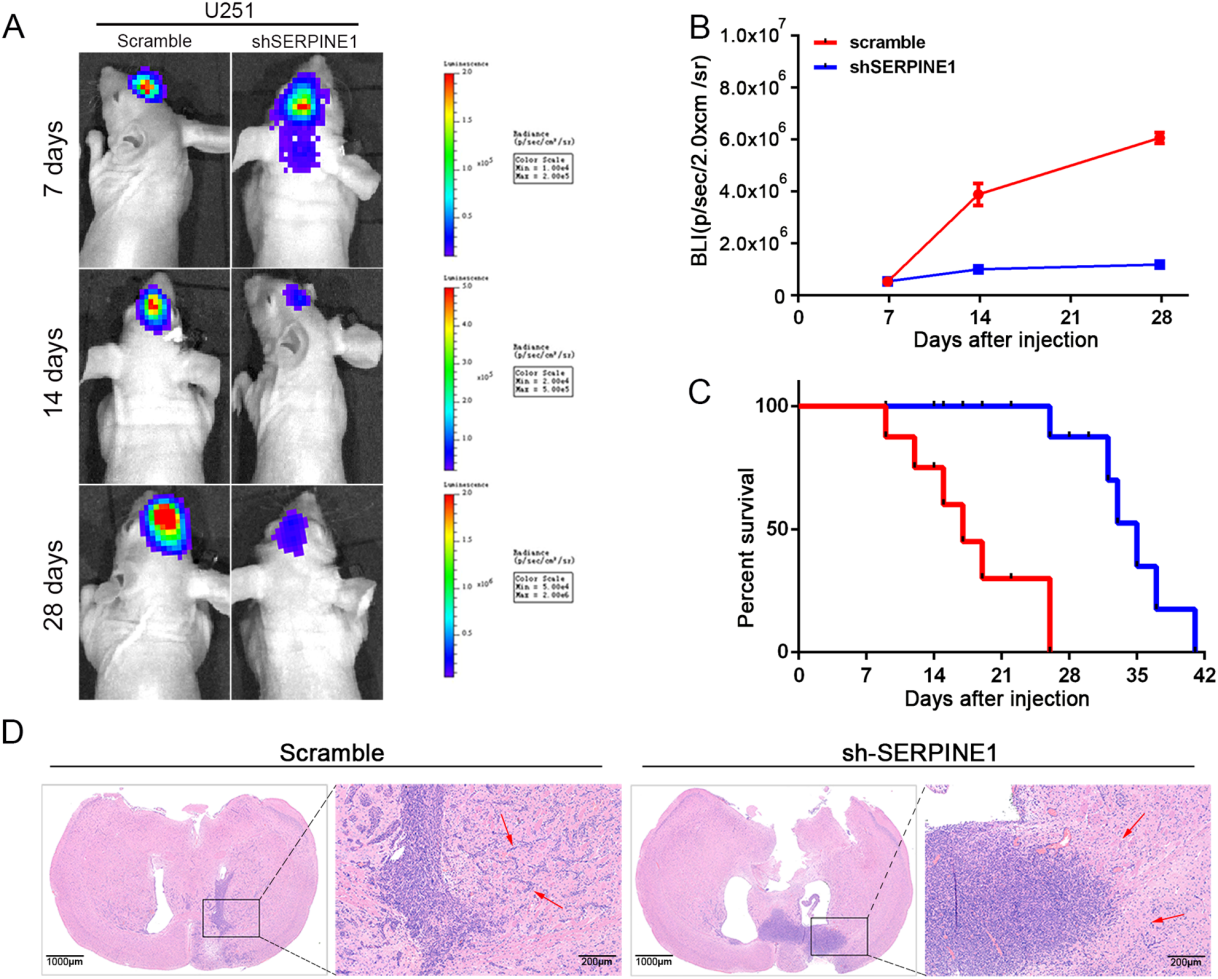
Effects of SERPINE1 knockdown on GBM cell invasion and infiltration in an orthotopic tumor model. **(A)** Representative bioluminescence images of mice on days 7, 14, and 28 after implantation of different cells; **(B)** Quantitative analysis of bioluminescence images for each group; **(C)** Survival of nude mice after serpine1 treatment; **(D)** HE staining of tumor sections, with red arrows indicating the peritumoral invasion and infiltration regions (×40, scale bar = 200 μm).

In *in vivo* experiments, tumor cell infiltration into surrounding tissues reflects the invasive characteristics of tumors. To investigate the effect of SERPINE1 on the invasive properties of GBM cells, a brain orthotopic xenograft model was established by implanting primary cells with stable SERPINE1 knockdown lentivirus into the mouse brain. Starting from 7 days after implantation, tumor proliferation was monitored using *in vivo* imaging on days 14 and 28. Bioluminescence imaging and HE staining showed that in the shSERPINE1 group, GBM cell proliferation and infiltration into surrounding tissues were significantly reduced compared to the scramble group (**Figures 5A–D**). These results suggest that inhibiting SERPINE1 effectively decreased the proliferation and invasiveness of GBM cells.

### 3.5 NR4A1 activates the JAK/STAT pathway after binding with SERPINE1

**Figure 6:**
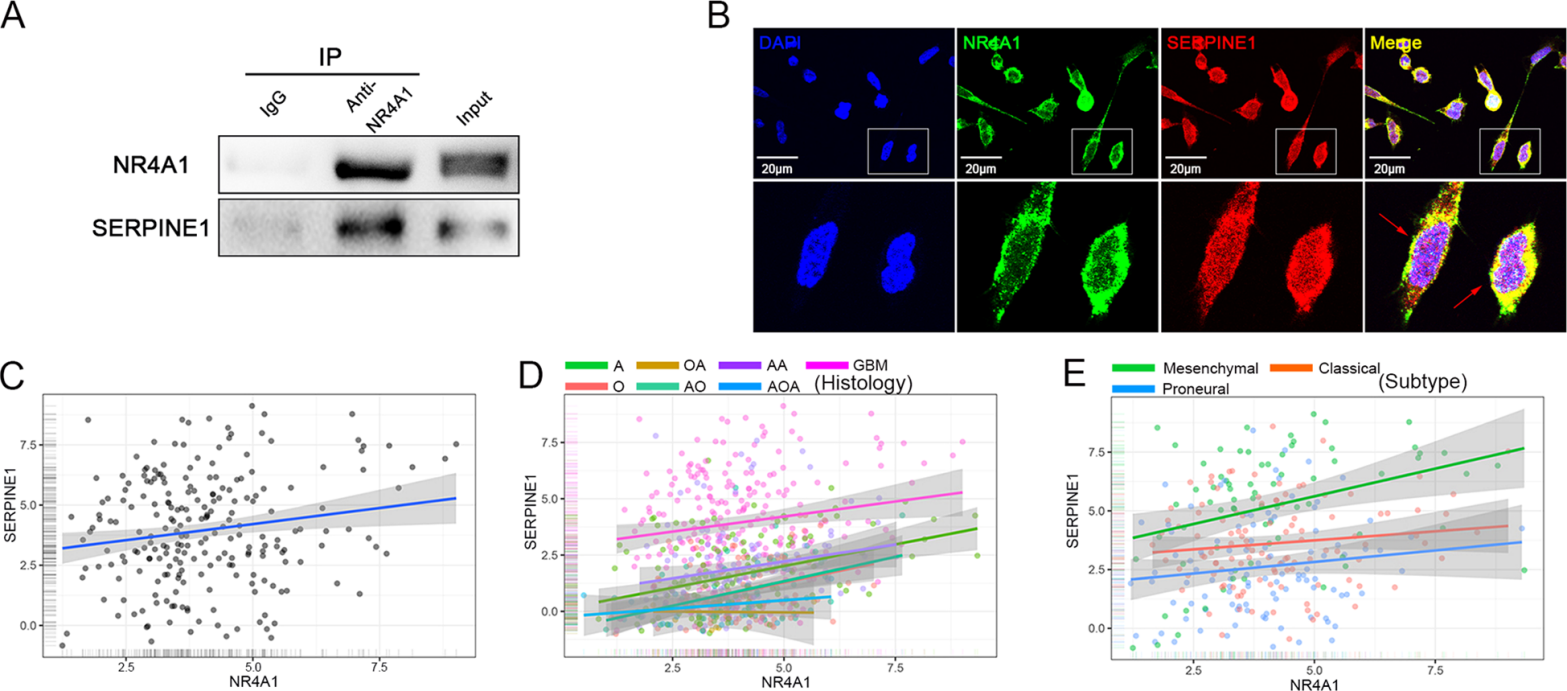
NR4A1 binds to SERPINE1 and shows expression correlation. **(A)** Immunoprecipitation assay was used to detect whether NR4A1 binds to the SERPINE1 protein in U251 cell; **(B)** Confocal microscopy was used to detect the colocalization of NR4A1 and SERPINE1 in the perinuclear region, with the lower image showing a magnified view of the white box in the upper image. The colocalization site is indicated by a red arrow, scale bar = 20 μm; **(C-E)** Bioinformatics analysis based on TCGA and CGGA databases was used to examine the expression correlation between NR4A1 and SERPINE1.

Through immunofluorescence co-localization results, we found that NR4A1 binds with SERPINE1 in the perinuclear region (**Figure 6B**). In NR4A1 immunoprecipitation experiments, we also confirmed the interaction between the two (**Figure 6A**). Additionally, we observed that after knockdown of NR4A1, the expression of SERPINE1 was reduced. Bioinformatics analysis also supported our conclusion, showing a correlation between the expression of NR4A1 and SERPINE1 (**Figures 6C–E**).

## 4 Discussion

In this study, combining PPI protein network analysis from the EMT database, we identified a novel NR4A1/SERPINE1 signaling axis in GBM cells. We found that SERPINE1 can regulate EMT in GBM cells. Interestingly, SERPINE1 may be regulated by NR4A1. These findings reveal a new approach to preventing the invasiveness of GBM cells in the hypoxic microenvironment by targeting the NR4A1/SERPINE1 axis in GBM.

The abnormal reactivation of EMT is associated with the malignant characteristics of tumor cells in cancer progression and metastasis, including promoting migration and invasion, increasing tumor stemness, and enhancing resistance to chemotherapy and immunotherapy (23). EMT is tightly regulated by a complex network composed of various intrinsic and extrinsic factors, including multiple transcription factors, post-translational controls, epigenetic modifications, and regulation mediated by non-coding RNAs (24). In this study, we first screened EMT-related genes in the EMT sample database. Through the analysis of differential mRNA expressions, prognosis, risk prediction, and other indicators, combined with PPI protein network analysis, we found that SERPINE1 may be a key gene regulating EMT in glioblastoma. Some classic studies have also confirmed the key regulatory role of SERPINE1 in tumor EMT: circFNDC3B drives EMT and metastasis in oral squamous cell carcinoma cells by regulating SERPINE1 (25). Similar studies involving circRNAs and SERPINE1 have also been conducted in glioma, but the underlying molecular mechanisms have not been thoroughly explored (26).

TCGA (The Cancer Genome Atlas) and CGGA (Chinese Glioma Genome Atlas) are two widely used genomic databases in cancer research, providing extensive genomic data on various cancer types, including glioblastoma multiforme (GBM). Their applications are primarily reflected in gene expression analysis, prognostic analysis, molecular subtyping, subtype identification, and multi-omics data integration for drug sensitivity. In this study, we performed expression analysis of SERPINE1 for prognosis and molecular subtyping and found that SERPINE1 is highly expressed in GBM, with the highest expression in the mesenchymal subtype, indicating a strong correlation between SERPINE1 and GBM invasion. Similarly, in breast cancer, similar studies have shown that PTX can counteract the expression of EMT and SERPINE1 in cervical cancer cells by reducing NF-kB.

The hypoxic microenvironment within tumors is one of the critical factors driving the enhanced invasiveness of tumor cells. During tumor growth, rapid proliferation often outpaces the blood vessel supply, leading to localized hypoxia (27). Hypoxia activates a series of molecular mechanisms that promote tumor cell invasion and metastasis. Our experimental results demonstrate that hypoxia enhances the invasive properties of glioblastoma (GBM) primary cells. Notably, we observed a compelling phenomenon: even under hypoxic conditions, inhibition of SERPINE1 effectively suppressed the invasiveness of GBM cells. Current studies have reported similar findings, indicating that hypoxia-induced reactive oxygen species (ROS) enhance hypoxic adaptation in glioblastoma by driving the HIF-1α-SERPINE1 signaling pathway (28). These findings underscore the pivotal role of SERPINE1 in the hypoxic microenvironment of GBM cells. Elucidating the upstream and downstream regulatory mechanisms of SERPINE1 now represents a pressing challenge in this field.

Our PPI protein network analysis suggests a potential interaction between SERPINE1 and NR4A1 proteins. Preliminary experiments indicate that NR4A1 may regulate SERPINE1 expression by binding to the SERPINE1 protein. Several studies support our conclusion: the binding of NR4A1 to proteins may alter their functions. For example, NR4A1 binding to Bcl-2 induces a conformational change in the Bcl-2 molecule, exposing its BH3 domain. This conformational change prevents Bcl-2 from inhibiting the pro-apoptotic proteins Bax and Bak, while inhibiting the anti-apoptotic protein Bcl-xL, thereby converting Bcl-2 from an anti-apoptotic protein to a pro-apoptotic one (29). This suggests that NR4A1 exerts its regulatory function by binding to proteins. Other studies also indicate that NR4A1 primarily binds to DNA response elements through its central DNA-binding domain, thereby regulating the transcription and expression of downstream genes (30). Additionally, NR4A1 can influence the transcriptional activation activity of other transcription factors through interaction. Therefore, NR4A1 exhibits versatility in regulating proteins or genes.

We evaluated the effect of SERPINE1 on the proliferation and invasion of GBM cells using an intracranial orthotopic tumor model. We found that after knocking down SERPINE1, the tumor size showed a trend of slower proliferation. Subsequent mouse brain tissue sections also indicated that inhibiting SERPINE1 effectively suppressed the spreading of GBM cells to the surrounding areas. Studies have reported the *in vitro* effects of the SERPINE1 inhibitor Tiplaxtinin in gliomas, but there is no application of this inhibitor in glioma *in vivo* models, which may be related to the drug’s permeability across the blood-brain barrier (31).

Here, we have highlighted some limitations of this study. The study did not include experiments on SERPINE1 inhibitors, nor did it involve corresponding *in vivo* drug experiments, which require further investigation in the future. We also neglected the restoration experiments on the relationship between NR4A1 and SERPINE1, and we hope to address this in future research.

## 5 Conclusion

Our research supports a novel therapeutic strategy for glioma. Our findings suggest that NR4A1 may regulate the protein expression of SERPINE1 by binding to it, and that inhibiting SERPINE1 can downregulate the invasion and infiltrative spread of glioma primary cells. This could represent a promising approach to suppress GBM invasion. Therefore, future studies should focus on the molecular targets and/or signaling pathways that target NR4A1 and SERPINE1, as they may contribute to the development of effective therapies for GBM.

## Data Availability

Publicly available datasets were analyzed in this study. This data can be found here: https://www.cancer.gov/ccg/research/genome-sequencing/tcga; https://www.cgga.org.cn/; https://xena.ucsc.edu/.

## References

[1] Mellinghoff IK Lu M Wen PY Taylor JW Maher EA Arrillaga-Romany I . Vorasidenib and ivosidenib in IDH1-mutant low-grade glioma: a randomized, perioperative phase 1 trial. Nat Med. (2023) 29:615–22. doi: 10.1038/s41591-022-02141-2, PMID: 36823302 PMC10313524

[2] Bruschi M Midjek L Ajlil Y Vairy S Lancien M Ghermaoui S . Diffuse midline glioma invasion and metastasis rely on cell-autonomous signaling. Neuro Oncol. (2024) 26:553–68. doi: 10.1093/neuonc/noad161, PMID: 37702430 PMC10912010

[3] Weller M Wen PY Chang SM Dirven L Lim M Monje M . Glioma. Nat Rev Dis Primers. (2024) 10:33. doi: 10.1038/s41572-024-00516-y, PMID: 38724526

[4] Varn FS Johnson KC Martinek J Huse JT Nasrallah MP Wesseling P . Glioma progression is shaped by genetic evolution and microenvironment interactions. Cell. (2022) 185:2184–99.e16. doi: 10.1016/j.cell.2022.04.038, PMID: 35649412 PMC9189056

[5] Jayaram MA Phillips JJ . Role of the microenvironment in glioma pathogenesis. Annu Rev Pathol. (2024) 19:181–201. doi: 10.1146/annurev-pathmechdis-051122-110348, PMID: 37832944

[6] Barthel L Hadamitzky M Dammann P Schedlowski M Sure U Thakur BK . Glioma: molecular signature and crossroads with tumor microenvironment. Cancer Metastasis Rev. (2022) 41:53–75. doi: 10.1007/s10555-021-09997-9, PMID: 34687436 PMC8924130

[7] Xue Z Liu J Xing W Mu F Wu Y Zhao J . Hypoxic glioma-derived exosomal miR-25-3p promotes macrophage M2 polarization by activating the PI3K-AKT-mTOR signaling pathway. J Nanobiotechnol. (2024) 22:628. doi: 10.1186/s12951-024-02888-5, PMID: 39407269 PMC11481566

[8] Ding XC Wang LL Zhang XD Xu JL Li PF Liang H . The relationship between expression of PD-L1 and HIF-1α in glioma cells under hypoxia. J Hematol Oncol. (2021) 14:92. doi: 10.1186/s13045-021-01102-5, PMID: 34118979 PMC8199387

[9] Chen Z Wang J Peng P Liu G Dong M Zhang X . Hypoxia-induced TGFBI maintains glioma stem cells by stabilizing EphA2. Theranostics. (2024) 14:5778–92. doi: 10.7150/thno.95141, PMID: 39346536 PMC11426234

[10] Saitoh M . Transcriptional regulation of EMT transcription factors in cancer. Semin Cancer Biol. (2023) 97:21–9. doi: 10.1016/j.semcancer.2023.10.001, PMID: 37802266

[11] Mao D Xu R Chen H Chen X Li D Song S . Cross-talk of focal adhesion-related gene defines prognosis and the immune microenvironment in gastric cancer. Front Cell Dev Biol. (2021) 9:716461. doi: 10.3389/fcell.2021.716461, PMID: 34660578 PMC8517448

[12] Wang HY Zhang XP Wang W . Regulation of epithelial-to-mesenchymal transition in hypoxia by the HIF-1α network. FEBS Lett. (2022) 596:338–49. doi: 10.1002/1873-3468.14258, PMID: 34905218

[13] Evans CE . Hypoxia-inducible factor signaling in inflammatory lung injury and repair. Cells. (2022) 11. doi: 10.3390/cells11020183, PMID: 35053299 PMC8774273

[14] Wang EL Zhang JJ Luo FM Fu MY Li D Peng J . Cerebellin-2 promotes endothelial-mesenchymal transition in hypoxic pulmonary hypertension rats by activating NF-κB/HIF-1α/Twist1 pathway. Life Sci. (2023) 328:121879. doi: 10.1016/j.lfs.2023.121879, PMID: 37355224

[15] Wu SM Jan YJ Tsai SC Pan HC Shen CC Yang CN . Targeting histone deacetylase-3 blocked epithelial-mesenchymal plasticity and metastatic dissemination in gastric cancer. Cell Biol Toxicol. (2023) 39:1873–96. doi: 10.1007/s10565-021-09673-2, PMID: 34973135 PMC10547655

[16] Zhang D Zhang JW Xu H Chen X Gao Y Jiang HG . Therapy-induced senescent tumor cell-derived extracellular vesicles promote colorectal cancer progression through SERPINE1-mediated NF-κB p65 nuclear translocation. Mol Cancer. (2024) 23:70. doi: 10.1186/s12943-024-01985-1, PMID: 38576002 PMC10993572

[17] Ju Y Wang Z Wang Q Jin S Sun P Wei Y . Pan-cancer analysis of SERPINE1 with a concentration on immune therapeutic and prognostic in gastric cancer. J Cell Mol Med. (2024) 28:e18579. doi: 10.1111/jcmm.18579, PMID: 39086142 PMC11291546

[18] Yang JD Ma L Zhu Z . SERPINE1 as a cancer-promoting gene in gastric adenocarcinoma: facilitates tumour cell proliferation, migration, and invasion by regulating EMT. J Chemother. (2019) 31:408–18. doi: 10.1080/1120009X.2019.1687996, PMID: 31724495

[19] Zhai Y Liu X Huang Z Zhang J Stalin A Tan Y . Data mining combines bioinformatics discover immunoinfiltration-related gene SERPINE1 as a biomarker for diagnosis and prognosis of stomach adenocarcinoma. Sci Rep. (2023) 13:1373. doi: 10.1038/s41598-023-28234-7, PMID: 36697459 PMC9876925

[20] Guo X Zhou H Liu Y Xu W Kanwore K Zhang L . Glial-cell-line-derived neurotrophic factor promotes glioblastoma cell migration and invasion via the SMAD2/3-SERPINE1-signaling axis. Int J Mol Sci. (2024) 25. doi: 10.3390/ijms251810229, PMID: 39337713 PMC11432670

[21] Xi X Liu N Wang Q Chu Y Yin Z Ding Y . ACT001, a novel PAI-1 inhibitor, exerts synergistic effects in combination with cisplatin by inhibiting PI3K/AKT pathway in glioma. Cell Death Dis. (2019) 10:757. doi: 10.1038/s41419-019-1986-2, PMID: 31591377 PMC6779874

[22] Zhang W Yan Z Zhao F He Q Xu H . TGF-β Score based on Silico Analysis can Robustly Predict Prognosis and Immunological Characteristics in Lower-grade Glioma: The Evidence from Multicenter Studies. Recent Pat Anticancer Drug Discov. (2024) 19:610–21. doi: 10.2174/1574892819666230915143632, PMID: 37718518

[23] Chen B Kiang KM Liu F Li C Li X Weiwei C . Neutrophil extracellular trap reprograms cancer metabolism to form a metastatic niche promoting non-small cell lung cancer brain metastasis. Adv Sci (Weinh). (2025):e08478. doi: 10.1002/advs.202508478, PMID: 41250997 PMC12850224

[24] Xiao S Tian L Gan X Xu X Liao M Song D . Role of ubiquitin-regulated EMT in cancer metastasis and chemoresistance. Int J Biol Sci. (2025) 21:6081–112. doi: 10.7150/ijbs.115401, PMID: 41208892 PMC12594574

[25] Li X Wang C Zhang H Li Y Hou D Liu D . circFNDC3B accelerates vasculature formation and metastasis in oral squamous cell carcinoma. Cancer Res. (2023) 83:1459–75. doi: 10.1158/0008-5472.CAN-22-2585, PMID: 36811957 PMC10152237

[26] Liu L Xiao S Wang Y Zhu Z Cao Y Yang S . Identification of a novel circular RNA circZNF652/miR-486-5p/SERPINE1 signaling cascade that regulates cancer aggressiveness in glioblastoma (GBM). Bioengineered. (2022) 13:1411–23. doi: 10.1080/21655979.2021.2018096, PMID: 35258403 PMC8805984

[27] Bemidinezhad A Al-Baghdadi A Alsarray A Abolhassani Y Kenjayev Y Gheybi F . Enhancing radiotherapy for hypoxic tumors: integrative strategies using bacteria and nanoparticles. IET Nanobiotechnol. (2025) 2025:2687439. doi: 10.1049/nbt2/2687439, PMID: 41246256 PMC12618120

[28] Zhang L Cao Y Guo X Wang X Han X Kanwore K . Hypoxia-induced ROS aggravate tumor progression through HIF-1α-SERPINE1 signaling in glioblastoma. J Zhejiang Univ Sci B. (2023) 24:32–49. doi: 10.1631/jzus.B2200269, PMID: 36632749 PMC9837376

[29] Safe S Karki K . The paradoxical roles of orphan nuclear receptor 4A (NR4A) in cancer. Mol Cancer Res. (2021) 19:180–91. doi: 10.1158/1541-7786.MCR-20-0707, PMID: 33106376 PMC7864866

[30] Pu ZQ Yu TF Liu D Jin CW Sadiq E Qiao X . NR4A1 enhances MKP7 expression to diminish JNK activation induced by ROS or ER-stress in pancreatic β cells for surviving. Cell Death Discov. (2021) 7:133. doi: 10.1038/s41420-021-00521-0, PMID: 34088892 PMC8178316

[31] Tatsuoka J Sano E Hanashima Y Yagi C Yamamuro S Sumi K . Anti-tumor effects of perampanel in Malignant glioma cells. Oncol Lett. (2022) 24:421. doi: 10.3892/ol.2022.13541, PMID: 36284648 PMC9580249

